# Genetic Sequence Variation in the *Plasmodium falciparum* Histidine-Rich Protein 2 Gene from Field Isolates in Tanzania: Impact on Malaria Rapid Diagnosis

**DOI:** 10.3390/genes13091642

**Published:** 2022-09-13

**Authors:** Robert D. Kaaya, Caroline Amour, Johnson J. Matowo, Franklin W. Mosha, Reginald A. Kavishe, Khalid B. Beshir

**Affiliations:** 1 Faculty of Medicine, Kilimanjaro Christian Medical University College, Moshi P.O. Box 2240, Tanzania; 2 Pan-African Malaria Vector Research Consortium, Moshi P.O. Box 2240, Tanzania; 3 Faculty of Infectious and Tropical Diseases, London School of Hygiene and Tropical Medicine, Keppel Street, London WC1E 7HT, UK

**Keywords:** malaria diagnosis, *Pfhrp2*, amino acid repeats, sequence variation, genetic polymorphism, *Plasmodium falciparum*

## Abstract

Malaria rapid diagnosis test (RDT) is crucial for managing the disease, and the effectiveness of detection depends on parameters such as sensitivity and specificity of the RDT. Several factors can affect the performance of RDT. In this study, we focused on the *pfhrp2* sequence variation and its impact on RDTs targeted by antigens encoded by *Plasmodium falciparum histidine-rich protein 2* (*pfhrp2)*. Field samples collected during cross-sectional surveys in Tanzania were sequenced to investigate the *pfhrp2* sequence diversity and evaluate the impact on HRP2-based RDT performance. We observed significant mean differences in amino acid repeats between current and previous studies. Several new amino acid repeats were found to occur at different frequencies, including types AAY, AHHAHHAAN, and AHHAA. Based on the abundance of types 2 and 7 amino acid repeats, the binary predictive model was able to predict RDT insensitivity by about 69% in the study area. About 85% of the major epitopes targeted by monoclonal antibodies (MAbs) in RDT were identified. Our study suggested that the extensive sequence variation in *pfhrp2* can contribute to reduced RDT sensitivity. The correlation between the different combinations of amino acid repeats and the performance of RDT in different malaria transmission settings should be investigated further.

## 1. Introduction

Malaria control and elimination largely depend on prompt and accurate diagnosis for effective treatment [1]. Since its inception in the early 1990s, point-of-care diagnosis proved to be reliable in malaria diagnosis in most parts of the world [2,3]. There has been a steady rise in demand and supply of test kits over the last 20 years [4]. There were approximately 348 million malaria rapid diagnostic test kits sold in 2019 by several companies [5]. The sub-Saharan African region (SSA) received about 80% of all RDT kits globally distributed, with more than 25 million (7%) of those kits distributed in Tanzania [5].

There is increasing evidence of *Plasmodium falciparum* lacking the hrp2/3 gene, enabling it to evade detection by HRP2-based RDTs. A study from Eritrea indicated that *pfhrp2/3* deletions are prevalent at 80.8% and 92.3%, respectively, and that prompted the switch to non-HRP2 RDTs [6]. Studies conducted in Tanzania have shown no *pfhrp2/3* deletion in some areas [7,8], but a low percentage has been reported in other parts of the country [9,10]. In regards to *pfhrp2/3* deletions, false positivity by RDT is a challenge that can result in the underestimation of the deletions.

The diagnostic coverage of RDT in Tanzania is around 90% in public and private health facilities replacing microscopy, which is only used in about 10% of all health facilities [11]. Most of the available RDT kits are based on histidine-rich protein 2 (HRP2), which is specific for detecting *P. falciparum,* a predominant parasite in Tanzania [5,12,13,14].

PFHRP2 is a 60–105 kDa water-soluble protein secreted by *P. falciparum* trophozoites and schizonts [15,16,17]. Approximately 2 hours after an infection, it is synthesized and secreted in the human host [18]. Gene encoding for this subtelomeric protein is located at positions 1374236 to 1375299 on chromosome 8 [19]. *pfhrp2* has a length of 1063 bp and consists of two exon (coding) regions and an intron (non-coding) region. The gene is flanked by four upstream and three downstream microsatellites [20,21].

The *pfhrp2* subtelomeric coding region is prone to chromosomal rearrangements with nine gene breaking points, making it highly polymorphic [22]. A large region of tandem repeats within the *pfhrp2* sequence encodes a polypeptide containing histidine, alanine, and aspartic acid. RDT detection panels include monoclonal antibodies (MAbs), which target specific HRP2 antigen epitopes [16,20]. There are about 13 major epitopes targeted by different monoclonal antibodies impregnated in the flow panel of RDT cassettes [23,24]. Detection sensitivity correlates well with the frequency and abundance of epitopes present in the sample. With the amino acid repetitive rearrangement in the *pfhrp2* region, partial epitopes can exist that are less reactive with capture antibodies than full-length epitopes [23].

A previous study by Baker et al. [20] classified the amino acid sequence of PfHRP2 into 24 repeat types. Type 2 (AHHAHHAD) and type 7 (AHHAAD) occur in high frequency (100%), and type 2 is associated with the basic function of the protein [25,26,27]. Based on the frequency of types 2 and 7 repeats, a prediction regression model was developed to estimate the sensitivity of RDT kits [28]. The model predicted that with parasitaemia ≤ 250 parasites/µL and the function of frequency between types 2 and 7 ˂ 43, HRP2-based RDT will fail to detect *P. falciparum* [28]. However, the model could not be reproduced 5 years later when its prediction did not match the WHO lot testing results set at >200 parasites/µL [27]. Several studies have shown that the sequence variation in *pfhrp2,* which leads to extensive epitope modification, might affect the performance of RDTs [24,28]. 

In light of *pfhrp2* deletions and sequence variations [9,29], the WHO recommends the systematic surveillance of RDT performance in areas with a high coverage of HRP2-based test kits [30]. This study investigated the natural amino acid sequence variation in *P. falciparum* field isolates to assess the performance of RDTs.

## 2. Materials and Methods

### 2.1. Study Areas and Samples

The samples used in this study were collected during community-based cross-sectional surveys in the long rainy season between April and June 2018 in Handeni and Moshi, north-eastern Tanzania. Community sensitization and engagement were carried out, and only participants who voluntarily consented to participate were enrolled. Handeni is characterized as a moderate–high malaria transmission area, whereas Moshi is a low malaria-endemic area [31,32].

### 2.2. Plasmodium Falciparum Detection

Dried blood samples were shipped to The London School of Hygiene and Tropical Medicine (LSHTM), where DNA extraction was carried out using a robotic DNA extraction system (Qiasymphony, QIAGEN, Hilden, Germany) [10,29,33]. A nested polymerase chain reaction (PCR) using specific primers for *P. falciparum* amplifying a fragment of 206 bp was performed as described elsewhere [34].

### 2.3. Pfhrp2 Exon 2 Amplification and Sequencing

*Pfhrp2* exon 2 was amplified with primers *Pfhrp2*-F1 (5′-CAAAAGGACTTAATTTAAATAAGAG-3′) and Pfhrp2-R1 (5′-AATAAATTTAATGGCGTAGGCA-3′). We employed semi-nested PCR using primer pairs *Pfhrp2*-F2 _5′-ATTATTACACGAAACTCAGCCAG-3′ and *Pfhrp2*-R1 _5′-AATAAATTTAATTGGCGTAGGCA-3′), designed to amplify *pfhrp2* exon 2 from filter papers, to assure sensitivity with an expected band size of 400–1050 bp [28]. PCR amplicon purification and sequencing were performed based on a previously published protocol [35].

### 2.4. Sequence Data Analysis

We used Geneious (Biomatters, San Diego, CA, USA) to conduct sequence analysis, including DNA quality check and translation into amino acid. Repeat pattern frequency and sequence length were analysed using R studio.

### 2.5. Statistical Analysis

Samples with parasitaemia of more than 1000 p/µL were used for this analysis. HRP2-RDT sensitivity prediction was performed following the model developed by Baker et al. [28]. Four categories were established based on the score of the function of the frequency of types 2 and 7. HRP2-RDT will be very sensitive if the score of types 2 and 7 frequencies is >100, sensitive if the score is 50–100, borderline if the score is 44–49, and non-sensitive if the score is <43 [36].

Data were entered and analysed using SPSS version 20 (SPSS Inc. Chicago, IL, USA) and the computer program Excel (Microsoft Office Excel 2016). Results are presented in tables and graphs as absolute numbers (N) and percentage values (%). The median amino acid (aa) length was compared using the non-parametric Mann–Whitney U test. The median frequencies of aa were compared using Fisher’s exact test since the expected values were less than 10. A *p*-value less than 0.05 was considered significant.

## 3. Results

**Figure 1:**
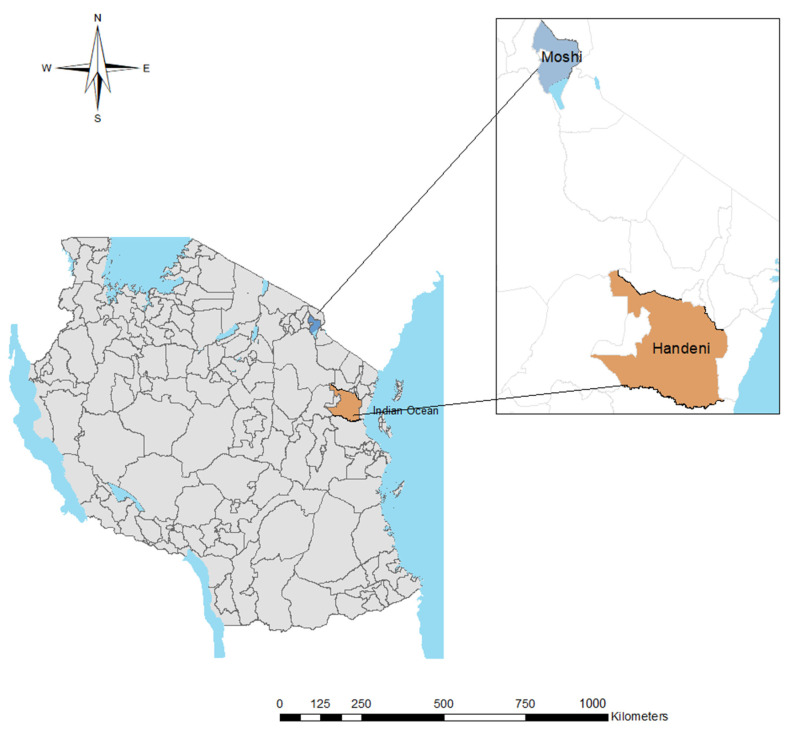
Map of Tanzania showing study sites (created by ArcGIS software v10.3, ESRI, Redlands, CA, USA).

The present study showed a sequence analysis of exon 2 of *pfhrp2* of 39 *P. falciparum* field isolates from Tanzania (Figure 1). The results of *Plasmodium* species identification in the study area have already been published elsewhere [24], and samples that were positive by RDT and microscopy (parasitaemia > 1000 p/µL) and identified as *P. falciparum* were selected for the direct sequencing. However, we were able to generate high-quality sequences in the samples from Handeni only probably due to the low levels of parasitaemia in the samples from Moshi.

The amino acid classification was carried out following the classification developed by Baker et al. [27]. Out of 24 amino acid repeat types, 15 were identified in this study, of which types 2 (AHHAHHAAD), 4 (AHH), and 7 (AHHAAD) were present in a high frequency (>89%) and abundance in all 39 samples. Types 10 (AHHAAAHHATD), 12 (AHHAAAHHEAATH), and 15 (AHHAHHAAN) were present in low frequency (2.6%) (Table 1).

### 3.1. Distribution of PfHRP2 Amino Acid Repeats in Tanzania

Our analysis of repeat amino acid sequence was compared with a previous study conducted in Tanzania in 2010 [27], and both studies analysed 39 samples. In about seven of the 24 types presented between the two studies, the mean number of amino acid repeats significantly differed (*p* < 0.05), whereas type 2 (AHHAHHAAD) more frequently occurred in all samples than the other types in the current study (Table 2).

### 3.2. HRP2-RDT Sensitivity Prediction in Detecting P. falciparum in Tanzania

RDT insensitivity was estimated to be 69% in detecting *P. falciparum* in the samples analysed using the Baker predictive model and sensitivity classification. The overall predicted sensitivity was 28%, and only 3% of the samples fell into the borderline sensitive group (Table 3).

### 3.3. Distribution of “Non-Baker” Amino Acid Repeats

**Figure 2:**
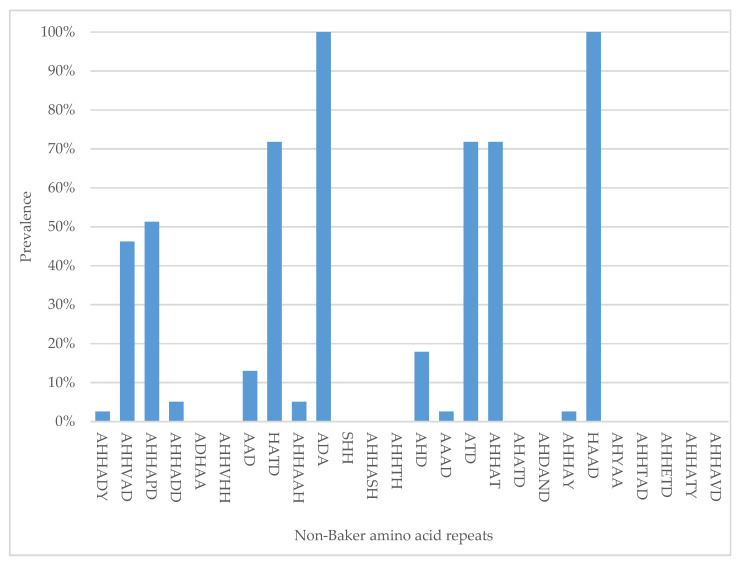
Frequency of “non-Baker” amino acid repeat types in 39 Tanzanian *P. falciparum* isolates.

The most prevalent types were ADA and HAAD occurring at 100% in all samples. Types AHHADY, AAAD, and AHHAY were the least prevalent (2.6%) (Figure 2).

### 3.4. RDT Major Epitopes in Tanzania

**Figure 3:**
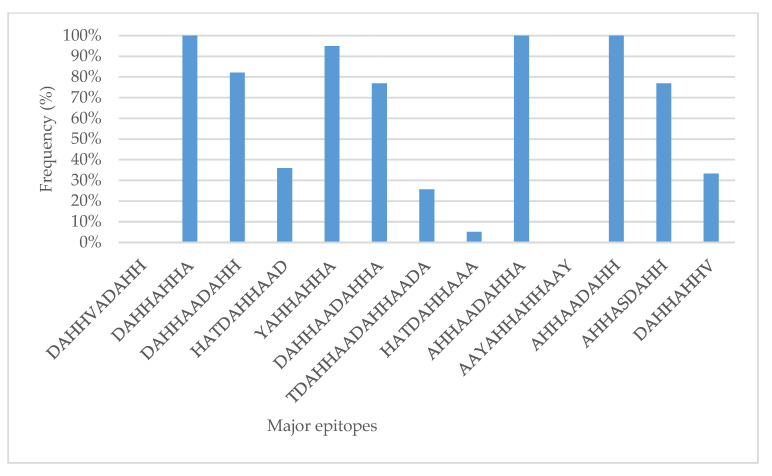
Frequency of *P. falciparum* HRP2 major epitopes in Tanzania.

There are about 13 major antigenic epitopes in PfHRP2 that are targeted by different classes of monoclonal antibodies (Mab) in HPR2-based RDTs. In the current study, 11 of the 13 (85%) were present. Epitopes such as DAHHAHHA, AHHAADAHHA, and AHHAADAHH that are targeted by 3A4/PTL-3, C1-13, and S2-5-C2-3 MAbs, respectively, were present in all samples (100%). Epitopes DAHHVADAHH and AAYAHHAHHAAY were not present in the field isolates in this study (Figure 3).

## 4. Discussion

*Pfhrp2* exon 2 sequences from the field isolates of *P. falciparum* showed substantial sequence diversity. We reported the sequence length, epitope type, and frequency and predicted the sensitivity of HRP2-RDT detection.

A total of 39 amino acid sequences were generated, ranging in length from 172 to 259 amino acids. The possible causes of the differences in length are frequent breaks and joining in chromosome 8 during meiosis and mitosis. The gene has about eight breaking points, and, every time, a new sequence is generated leading to the observed variation in length and arrangement [22,37,38,39]. Studies have demonstrated that this could be a normal mechanism in the parasite and ultimately can lead to polymorphism in the gene. In Tanzania, amino acid lengths ranging from 207 to 287 have been observed, which is also the case in the global range of amino acid lengths [27].

Following Baker’s amino acid classification, we reported the existence of 15 of 24 (62.5%) amino acid repeats, of which 12 repeats were also previously found in Tanzania [27]. Amino acid repeat types AAY, AHHAHHAAN, and AHHAA are new and hereby reported for the first time in the field isolates from Tanzania. Only one repeat type (ARHAAD) was previously reported but not in the current study [27]. It is argued that the recombination of polyclonal infection of *P. falciparum* particularly in high transmission areas can result in the diversity and emergence of different polymorphisms in the *pfhrp2* gene [27]. Several studies have demonstrated the possibility of reduced sensitivity and overall performance of RDT due to the sequence variation in the *pfhrp2* gene [24,28].

The results of the sequence analysis from this study showed that types 2 and 7 amino acid repeats are common in most samples, occurring at a high prevalence but at different frequencies. These two types are believed to form the basis of major epitopes, although the overall function of these repeats in the functional mechanism of HRP2 in *P. falciparum* is not known [40,41]. Different studies have shown a significant association between the frequency of the two types and the performance of RDT at different parasitaemia levels [28,42,43]. The results of our analysis based on a combined frequency between types 2 and 7 indicate that 69% of the samples had a score of ˂43 repeats. This score suggests a low frequency of types 2 and 7, which implies a predicted reduced sensitivity to RDT. This is in line with Baker’s regression model, which predicts RDT insensitivity, especially in low parasitaemia.

We also found 14 amino acid repeats that are not in Baker’s classification (non-Baker repeats). Types ADA and HAAD were present at relatively high proportions in all of the samples (100%), suggesting an important role in the physiological system mechanisms of the parasite; that is why it is expressed in high abundance. Studies in Madagascar and Papua New Guinea previously reported some of the non-Baker repeats but at much lower frequencies [26,44]. Their contribution to the efficacy and performance of RDT is yet to be determined, and this calls for further investigation.

In this study, we found 11 of the 13 (85%) major epitopes that are globally targeted by most of the distributed RDT kits. The most prevalent epitopes were DAHHAHHA, AHHAADAHHA, and AHHAADAHH, which were present in all isolates analysed. These findings indicate that RDT kits with monoclonal antibodies targeting these epitopes will optimally perform in the study area. Apparently, the three epitopes also occur in high proportions elsewhere in Africa [26]. Laboratory studies have tested the same MAbs in different field isolates and observed significant differences in reactivity, suggesting that sequence variation and frequency have an impact on RDT performance [23,24].

Genetic diversity in *pfhrp2* can potentially result in the expression of more or less complex PfHRP2. Previous studies have shown that high antibodies to PfHRP2 might lead to reduced sensitivity of RDTs, particularly in high transmission areas due to the formation of antibody–PfHPRP2 complexes making the protein unavailable in the plasma. The protein elicits antibodies with a short low half-life since there is no correlation between anti-PFHRP2 titres and the age of study participants [45].

Our study provided evidence of sequence variation in *pfhrp2* in the field samples for Tanzania. Comparing our results with a previous study, it is evident that there are significant differences in the amino acid repeats. We could not validate Baker’s model to explain the level of RDT performance in this study, but we predicted the effect of *pfhrp2* polymorphism on RDT sensitivity in Tanzania. More studies should focus on the correlation between RDT performance in relation to the amino acid repeat types of both “Baker” and “non-Baker”.

## 5. Conclusions

The findings from this study provided information on *pfhrp2* sequence polymorphism and predicted the effect on RDT performance. The data on antigenic epitopes presented in this study will inform on the purchase and supply of effective RDT in Tanzania. There is an urgent need to deploy a novel and unconventional point-of-care test that exploits magnetic resonance in malaria diagnosis [46,47].

## 6. Study Limitations

The limited number of samples analysed in this study might have underestimated the effect of amino acid repeats on RDT performance particularly in lower Moshi where malaria prevalence is very low. Recent data from the study areas could highlight a different amino acid repeat pattern. This study could not validate Baker’s model based on the field isolates from Tanzania, but it could predict that, in an event of low parasitaemia, RDT could be insensitive. We did not sequence *pfhrp3*, which is the isoform of *pfhrp2* and usually cross-reacts to anti-HRP2 and increases sensitivity to RDT.

## Figures and Tables

**Table 1:** Prevalence and occurrence of different amino acid repeats observed in *P. falciparum* HRP2 from field isolates in north-eastern Tanzania.

AA Code	AA Type	Occurrence	Frequency
TYPE 1	AHHAHHVAD	29	38.5%
TYPE 2	AHHAHHAAD	335	100%
TYPE 3	AHHAHHAAY	36	71.8%
TYPE 4	AHH	228	94.9%
TYPE 5	AHHAHHASD	35	76.9%
TYPE 6	AHHATD	50	69.2%
TYPE 7	AHHAAD	122	89.7%
TYPE 8	AHHAAY	32	66.7%
TYPE 9	AAY	2	5.1%
TYPE 10	AHHAAAHHATD	1	2.6%
TYPE 11	AHN	0	0%
TYPE 12	AHHAAAHHEAATH	1	2.6%
TYPE 13	AHHASD	2	5.1%
TYPE 14	AHHAHHATD	5	10.3%
TYPE 15	AHHAHHAAN	1	2.6%
TYPE 16	AHHAAN	0	0%
TYPE 17	AHHDG	0	0%
TYPE 18	AHHDD	0	0%
TYPE 19	AHHAA	18	41%
TYPE 20	SHHDD	0	0%
TYPE 21	AHHAHHATY	0	0%
TYPE 22	AHHAHHAGD	0	0%
TYPE 23	ARHAAD	0	0%
TYPE 24	AHHTHHAAD	0	0%

**Table 2:** Comparison of amino acid mean length and frequency of each repeat in PfHRP2 in parasites from previous and current studies in Tanzania.

Surveys	n	Length (aa)	Number of Individual Repeats																							
			1 *	2 *	3	4 *	5	6 *	7	8	9 *	10 *	11	12	13	14	15	16	17	18	19 *	20	21	22	23	24
**Global ^#^**	458	187–306	0–7	5–19	0–3	0–4	0–3	0–7	0–13	0–3	0–1	0–4	0–1	1	0–2	0–1	-	-	-	-	0–1	0–1	0–1	0–1	0–1	0–1
**Previous study ^#^**	39	207–287	0–7	8–17	0–2	0–2	0–2	2–6	2–9	0–3	0	0–3	0	1	0–1	0–1	-	-	-	-	0	0	0	0	0–1	0
**Current study**	39	173–260	0–5	3–12	0–2	0–20	0–2	0–3	0–9	0–2	0–1	0–1	0	0–1	0–1	0–2	0–1	0	0	0	0–3	0	0	0	0	0
**Mean**		232	0.7	8.6	0.9	5.8	0.9	1.3	3.1	0.8	0.05	0.02	0	0.03	0.05	0.1	0.03	0	0	0	0.5	0	0	0	0	0
**Median**		237	0	9	1	4	1	1	2	1	0	0	0	0	0	0	0	0	0	0	0	0	0	0	0	0

* Mean number of this repeat is significantly different from that in Baker et al.′s [27] study (*p* < 0.05), ^#^ [27].

**Table 3:** Prediction of RDT sensitivity in field isolates of *P. falciparum* in north-eastern Tanzania.

No	Sample	Type 2 (AHHAHHAAD)	Type 7 (AHHAAD)	Score (Type 2 × Type 7)	Sensitivity
1	B01_TZHRPR.ab1	11	1	11	Non-sensitive
2	B02_TZHRPR.ab1	9	1	9	Non-sensitive
3	B04_TZHRPR.ab1	9	1	9	Non-sensitive
4	B05_TZHRPR.ab1	4	3	12	Non-sensitive
5	B06_TZHRPR.ab1	10	6	60	Sensitive
6	B07_TZHRPR.ab1	9	1	9	Non-sensitive
7	B08_TZHRPR.ab1	9	5	45	Borderline
8	B11_TZHRPR.ab1	8	2	16	Non-sensitive
9	C01_TZHRPR.ab1	12	7	84	Sensitive
10	C02_TZHRPR.ab1	10	6	60	Sensitive
11	C03_TZHRPR.ab1	10	5	50	Sensitive
12	C04_TZHRPR.ab1	5	2	10	Non-sensitive
13	C06_TZHRPR.ab1	9	2	18	Non-sensitive
14	C07_TZHRPR.ab1	10	2	20	Non-sensitive
15	C08_TZHRPR.ab1	6	3	18	Non-sensitive
16	D01_TZHRPR.ab1	7	0	0	Non-sensitive
17	D03_TZHRPR.ab1	11	5	55	Sensitive
18	D07_TZHRPR.ab1	8	0	0	Non-sensitive
19	D11_TZHRPR.ab1	7	3	21	Non-sensitive
20	E01_TZHRPR.ab1	9	2	18	Non-sensitive
21	E02_TZHRPR.ab1	12	2	24	Non-sensitive
22	E03_TZHRPR.ab1	7	1	7	Non-sensitive
23	E04_TZHRPR.ab1	8	0	0	Non-sensitive
24	E05_TZHRPR.ab1	10	2	20	Non-sensitive
25	E06_TZHRPR.ab1	10	7	70	Sensitive
26	E07_TZHRPR.ab1	6	9	54	Sensitive
27	E08_TZHRPR.ab1	9	2	18	Non-sensitive
28	E11_TZHRPR.ab1	4	2	8	Non-sensitive
29	E12_TZHRPR.ab1	9	7	63	Sensitive
30	G02_TZHRPR.ab1	11	3	33	Non-sensitive
31	G03_TZHRPR.ab1	10	6	60	Sensitive
32	G06_TZHRPR.ab1	11	5	55	Sensitive
33	G07_TZHRPR.ab1	10	4	40	Non-sensitive
34	G11_TZHRPR.ab1	9	1	9	Non-sensitive
35	G12_TZHRPR.ab1	3	2	6	Non-sensitive
36	H02_TZHRPR.ab1	10	0	0	Non-sensitive
37	H03_TZHRPR.ab1	11	7	77	Sensitive
38	H06_TZHRPR.ab1	5	3	15	Non-sensitive
39	H07_TZHRPR.ab1	7	2	14	Non-sensitive

## Data Availability

Not applicable.
